# MccB‐catalyzed C‐terminal Thioesterification for Protein Bioconjugation

**DOI:** 10.1002/cpz1.70398

**Published:** 2026-06-11

**Authors:** Di Yang, Amy M. Weeks

**Affiliations:** ^1^ Department of Biochemistry University of Wisconsin‐Madison Madison WI USA

**Keywords:** expressed protein ligation, protein bioconjugation, protein C terminus, protein thioester, subtiligase

## Abstract

Protein bioconjugation enables the installation of functional groups, probes, and payloads that cannot be genetically encoded, thereby enabling the study and manipulation of biological systems. An important class of intermediates in protein bioconjugation is C‐terminal protein α‐thioesters, which are kinetically stable yet thermodynamically activated as electrophiles for C‐terminal functionalization. An ATP‐driven, MccB‐catalyzed enzymatic system for synthesis of protein C‐terminal thioesters was recently developed to complement existing tools for chemical and intein‐mediated synthesis of C‐terminal thioesters. This system relies on modification of the target protein with a thioesterification C‐terminal handle (TeCH tag) that is specifically recognized by MccB, enabling conversion of the C‐terminal α‐carboxylate to an α‐thioester. Thioesters generated with the MccB/TeCH tag system can be integrated with well‐established bioconjugation methods, including expressed protein ligation and enzyme‐catalyzed expressed protein ligation, expanding the scope and versatility of this approach. This article outlines the steps for applying the MccB/TeCH tag system for epitope‐specific C‐terminal thioesterification and for the use of MccB‐generated thioesters for expressed protein ligation and enzyme‐catalyzed expressed protein ligation. © 2026 The Author(s). *Current Protocols* published by Wiley Periodicals LLC.

**Basic Protocol 1**: MccB‐catalyzed protein C‐terminal thioesterification

**Alternate Protocol 1**: MccB‐catalyzed expressed protein ligation

**Alternate Protocol 2**: Stepwise MccB‐catalyzed protein C‐terminal thioesterification followed by expressed protein ligation

**Support Protocol 1**: Expression and purification of MccB

**Basic Protocol 2**: MccB‐ and subtiligase‐catalyzed expressed protein ligation

## INTRODUCTION

**Figure 1:**
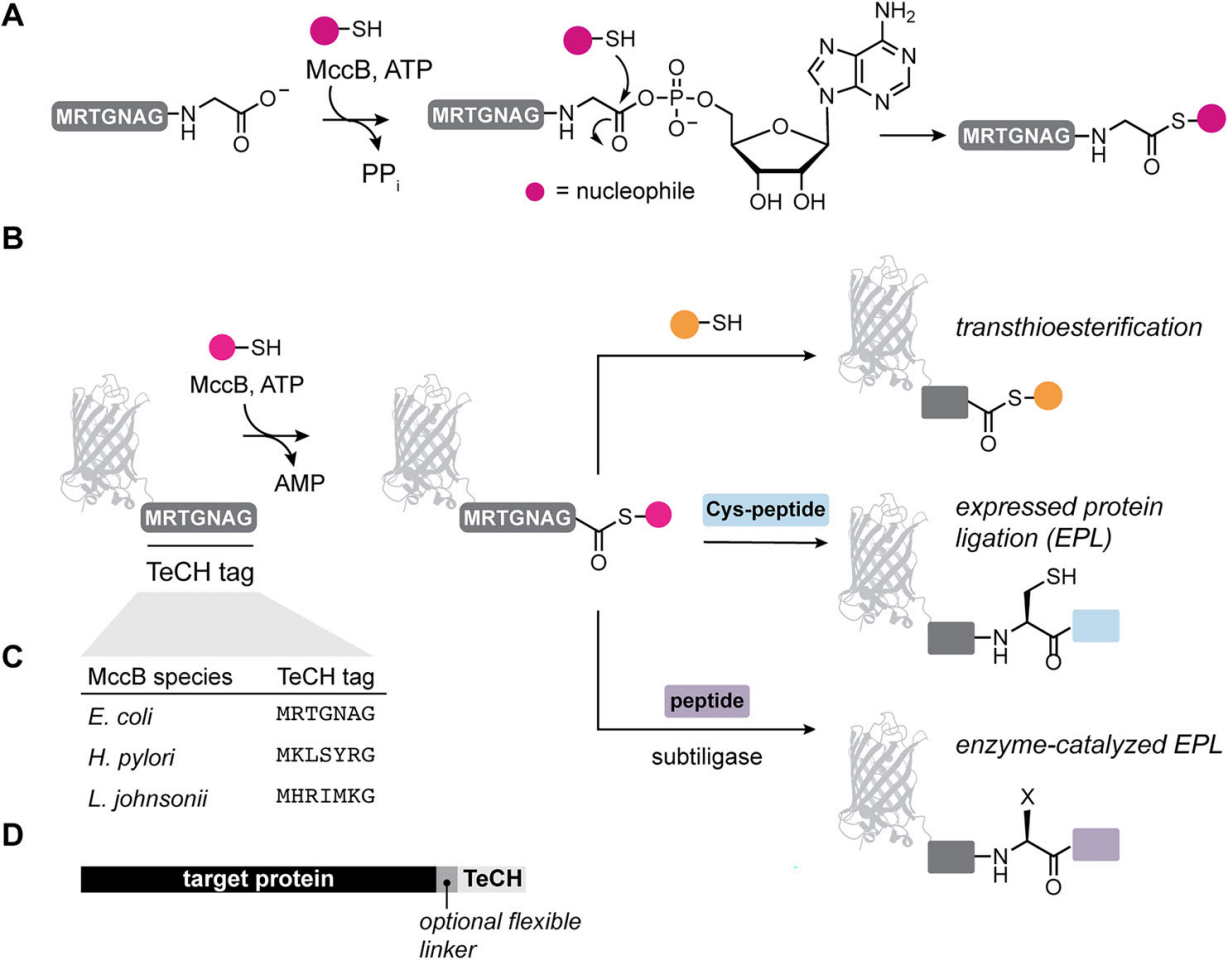
MccB‐catalyzed protein C‐terminal thioesterification and its applications. (A) ATP driven C‐terminal activation of MccA‐N7G (MRTGNAG) by *E. coli* MccB. (B) Protein C‐terminal modification strategies enabled by the MccB/TeCH tag system. (C) Cognate TeCH tag sequences for MccBs from different bacterial species. (D) Design of the target protein construct for application of the MccB/TeCH tag system for C‐terminal thioesterification.

Protein C‐terminal thioesters are key intermediates in protein semisynthesis and bioconjugation (Thompson & Muir, 2020). For example, native chemical ligation (NCL) (Dawson et al., 1994) and expressed protein ligation (EPL) (Muir et al., 1998) exploit these intermediates to install probes and payloads that cannot be genetically encoded, expanding the toolkit for investigating and manipulating biological systems. Until recently, direct synthesis of protein thioesters relied on a single approach, fusion of the protein of interest to an engineered intein (Shah & Muir, 2014). To address this limitation, we developed the MccB/TeCH tag system, which enables MccB‐catalyzed, ATP‐driven conversion of protein and peptide C termini to thioesters for use in NCL, EPL, and other bioconjugation reactions (Frazier et al., 2025) (**Figure** **1**). An advantage of the MccB/TeCH tag system is that, in contrast to intein chemistry, it relies on a multiturnover enzymatic reaction for thioester generation. MccB/TeCH tag can therefore be used in enzyme‐catalyzed EPL (Henager et al., 2016) for ATP‐dependent thioester regeneration, driving the reaction equilibrium toward the desired ligation product.

MccB is a member of the ThiF enzyme superfamily, which includes E1 ubiquitin‐activating enzymes as well as enzymes involved in cofactor and ribosomally produced and post‐translationally modified peptide (RiPP) biosynthesis (Burroughs et al., 2009; Hochstrasser, 2000). MccB's native function is in the biosynthesis of the RiPP natural product microcin C7 from the genetically encoded precursor peptide MccA (MRTGNAN) (Guijarro et al., 1995; Roush et al., 2008). MccB activates the C‐terminus of MccA by *O*‐AMPylation to generate an electrophilic intermediate that is intramolecularly captured by the C‐terminal Asn side chain. The resultant succinimide intermediate undergoes *N*‐AMPylation and rearrangement to form the N‐P bond observed in microcin C7 (Roush et al., 2008). We found that a non‐native substrate in which the C‐terminal Asn residue is substituted with Gly (MccA‐N7G) retains the ability to undergo MccB‐catalyzed *O*‐AMPylation to form an intermediate that can be intercepted by exogenously added thiol nucleophiles to form a C‐terminal thioester (Frazier et al., 2025) (**Figure** **1** **A**). MccA‐N7G, which we term the Thioesterification C‐terminal Handle, or TeCH tag, can be fused to the C‐terminus of a target protein to enable MccB‐catalyzed thioesterification.

Target proteins fused to the TeCH tag can undergo ATP‐dependent, MccB‐catalyzed C‐terminal thioesterification, producing an intermediate that can be integrated with the existing chemical biology toolbox for C‐terminal protein modification (**Figure** **1** **B**). For example, MccB‐generated protein C‐terminal thioesters can be used in EPL to synthesize N‐terminal Cys peptides or in subtiligase‐catalyzed EPL to ligate to diverse peptide sequences (Frazier et al., 2025; Henager et al., 2016; Muir et al., 1998). MccB‐catalyzed C‐terminal thioesterification reactions can also be performed in tandem with other bioconjugation methods for multi‐site modification of the target protein. For example, dual N‐ and C‐terminal labeling of a target protein can be achieved using MccB/subtiligase‐catalyzed EPL in combination with N‐terminal sortase modification in either a one‐pot or telescoped format (Frazier et al., 2025).

The high specificity of *E. coli* MccB for its cognate TeCH tag sequence enables epitope‐specific bioconjugation in complex mixtures. To expand the scope of available TeCH tag sequences, several other MccA/MccB pairs have been confirmed to function in the MccB/TeCH tag approach (Bantysh et al., 2014; Frazier et al., 2025) (**Figure** **1** **C**). In particular, MccA/MccB pairs from *Lactobacillus johnsonii*, *Helicobacter pylori*, and *Histophilus somni* were found to be orthogonal to *E. coli* MccA/MccB and to one another. Each of the MccA sequences can be converted to a TeCH tag by substituting the C‐terminal Asn with Gly. The identification of these orthogonal MccB/TeCH tag pairs expands the available sequences for generating C‐terminal thioesters and provides the option of performing orthogonal labeling of different proteins within a mixture.

This protocol describes how to use the MccB/TeCH tag system to generate protein C‐terminal thioesters for use in EPL and enzyme‐catalyzed EPL. The Strategic Planning section describes factors that should be considered in the design of an MccB‐catalyzed C‐terminal modification strategy, including fusion of the TeCH tag to the target protein, selection of MccB/TeCH tag homolog (**Figure** **1** **D**), and the reaction conditions to be used. Basic Protocol 1 describes how to perform MccB‐catalyzed C‐terminal thioesterification with sodium 2‐mercaptoethanesulfonate (Mesna) and, optionally, *trans*‐thioesterification with an alternative thiol nucleophile. Alternative protocols are provided for use when the desired modification is a peptide bond at the N‐terminal Cys of a peptide. This procedure can be performed in one pot (Alternate Protocol 1) or after isolation of the protein C‐terminal thioester (Alternate Protocol 2). Support Protocol 1 describes how to purify MccB homologs after recombinant expression in *E. coli*. Basic Protocol 2 describes how to use MccB in combination with subtiligase for enzyme‐catalyzed expressed protein ligation.

## STRATEGIC PLANNING

Before beginning Basic Protocol 1 of Basic Protocol 2, a key consideration is the introduction of the TeCH tag to the C‐terminus of the target protein (**Figure** **1** **D**). For MccB to efficiently convert the C‐terminus to a thioester, the TeCH tag must be accessible to MccB binding. If the native C terminus of the target protein is not sufficiently flexible to enable MccB activity when the TeCH tag is directly appended, a short linker (such as GGGG, GAGS, or GSGS) may be added between the target protein and the TeCH tag. For all proteins we have tested, a linker of four or fewer amino acids has been sufficient to enable robust MccB activity. Another important consideration is whether the C‐terminal linker‐TeCH tag sequence is subject to proteolysis during expression in *E. coli*. We have observed that for some proteins, the C‐terminal TeCH tag is partially proteolyzed during *E. coli* expression. It is therefore advisable to analyze the TeCH‐tagged target protein by high‐resolution intact protein LC‐MS to confirm that the TeCH tag is present before beginning.

A second consideration is choosing the MccB homolog and the cognate TeCH tag to use (**Figure** **1** **C**). Although all MccB/TeCH tag pairs that we have tested enable near‐complete conversion of the target protein to a C‐terminal thioester, *E. coli* MccB has several advantages if only one MccB/TeCH tag pair is needed. *E. coli* MccB can be expressed and purified at the highest yield of the homologs we have tested and does not require co‐expression with the GroEL/GroES chaperonin system. *E. coli* MccB also requires the lowest concentration of Mesna to give complete conversion to the C‐terminal thioester. However, if sequence considerations or the desire to modify multiple proteins in a single reaction mixture suggest that another MccB homolog is preferred, complete conversion is possible and expected when the reaction conditions are adjusted accordingly (see Basic Protocol 1).

Because the *O*‐AMPylated intermediate undergoes rapid hydrolysis, selecting an appropriate nucleophile is important for effectively capturing the activated species. We have found that sodium 2‐mercaptoethanesulfonate (Mesna) works very efficiently; however, other nucleophiles may also be suitable. Based on our studies, nucleophilic capture of the peptidyl‐*O*‐AMP species occurs within the enzyme active site, and larger nucleophiles that cannot access the active site effectively produce low yields or no conversion. If a C‐terminal thioester generated from a larger thiol nucleophile is desired, it is possible to effect non‐enzymatic *trans*‐thioesterification starting from the protein‐2‐mercaptoethanesulfonate (Mes) thioester, as described in Basic Protocol 1.

For Basic Protocol 2, an additional consideration is the selection of the N‐terminal sequence of the peptide to be ligated to the protein C‐terminal thioester. While subtiligase has broad specificity for N‐terminal sequences, certain sequences are less favorable (Weeks & Wells, 2018, 2020b). These include sequences with acidic residues or proline in either of the first two positions. If the ligation junction must include these sequences, variants of subtiligase with altered sequence specificity can be chosen (Weeks & Wells, 2018). Additional information on using subtiligase specificity variants for bioconjugation can be found in an earlier Current Protocols article (Weeks & Wells, 2020a).

For Basic Protocols 1 and 2, careful attention should be paid to the composition of the reaction buffer. Both MccB and subtiligase function efficiently at pH 8.0, and this reaction pH also enables downstream non‐enzymatic reactions such as *trans*‐thioesterification and *S*‐to‐*N* acyl transfer. MccB activity is compatible with both Tris and HEPES buffers. However, it is important to note that nucleophilic buffer components, such as Tris, can undergo subtiligase‐catalyzed ligation to the target protein's C‐terminus. In Basic Protocol 2, nucleophilic buffer components, such as Tris, glycerol, DTT, and β‐mercaptoethanol, should therefore be avoided. When reducing agents are required, tris(2‐carboxyethyl)phosphine (TCEP) is usually used because of its strong reducing power and low nucleophilicity.

*NOTE*: All protocols involving animals must be reviewed and approved by the appropriate Animal Care and Use Committee and must follow regulations for the care and use of laboratory animals. Appropriate informed consent is necessary for obtaining and use of human study material.

## Basic Protocol 1 MccB‐CATALYZED PROTEIN C‐TERMINAL THIOESTERIFICATION

**Figure 2:**
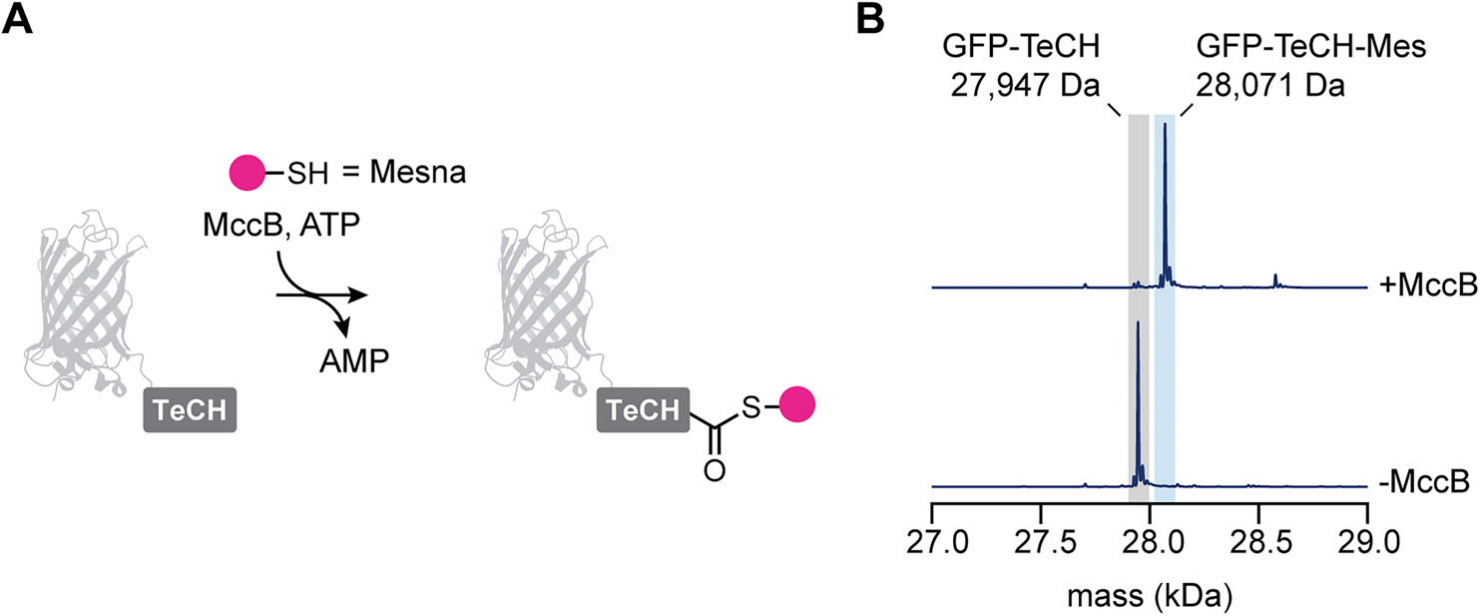
Example data for MccB‐catalyzed C‐terminal thioesterification. (A) Reaction scheme for MccB‐catalyzed C‐terminal thioesterification with Mesna. (B) TeCH‐tagged GFP (bottom) is converted to the corresponding Mes thioester (top) as shown by intact protein high‐resolution LC‐MS.

This protocol describes how to perform MccB‐catalyzed, ATP‐driven C‐terminal thioesterification of TeCH‐tagged peptides and proteins (Frazier et al., 2025). The reaction proceeds under mild conditions when the TeCH‐tagged target protein is incubated with MccB, ATP, and sodium 2‐mercaptoethanesulfonate (Mesna) or another suitable thiol. Optionally, transthioesterification can be performed to exchange the 2‐mercaptoethanesulfonate (Mes) thioester with an alternative thiol. Near‐quantitative conversion of the target protein to a C‐terminal thioester is typically achieved with this protocol (Figure 2).

### Materials

10× Reaction buffer (see recipe)
100 mM Adenosine 5′‐triphosphate (ATP), pH 7.0 (see recipe)
1 M Tris(2‐carboxyethyl)phosphine (TCEP), pH 8.0 (see recipe)
Ultrapure MilliQ water
500 mM Sodium 2‐mercaptoethanesulfonate (Mesna), pH 7.0 (prepare fresh; see recipe)
100 µM MccB (see Support Protocol 1)
C‐terminally TeCH‐tagged target protein (see Strategic Planning for buffer considerations)

0.5‐mL See Zeba Spin Desalting Column, 7K MWCO (Thermo Fisher Scientific, cat. no. 89882)
pH indicator strips (pH 0‐14, Sigma‐Aldrich, cat. no. 1095350001)
LC‐MS system suitable for intact protein analysis (e.g., Agilent 1290 LC system coupled to Agilent 6230 time‐of‐flight (TOF) mass spectrometer)
Analytical HPLC column suitable for intact protein analysis on the LC‐MS system (e.g., Agilent PLRP‐S 1000Å, 2.1 × 50 mm, 5 µm, cat. no. PL1912‐1502)
Software capable of deconvolution of multiply charged intact protein mass spectra (e.g., Agilent BioConfirm, Maximum Entropy algorithm)

1 Prepare the reaction mixture according to the table below.

**Table:** 

**Component**	**Stock concentration**	**Final concentration**	**Vol. for a 100 µL reaction**
Reaction buffer	10×	1×	10 µL
Mesna	500 mM	5 mM	1 µL
TCEP (optional)	1 M	5 mM	0.5 µL
Target protein	100 µM	50 µM	50 µL
ATP	100 mM	5 mM	5 µL
MccB	100 µM	5 µM	5 µL
Milli‐Q water	–	–	28.5 µL
  TCEP helps to maintain Mesna in its reduced form but is optional. Mix each component thoroughly by pipetting up and down after each step. If the pH of reagents used in the reaction has not been adjusted to neutral, it is advisable to verify that the pH of the reaction mixture is around 8.0 before adding MccB. MccB should be added last to initiate the reaction. While 100 µL is a typical reaction volume, the reaction can be scaled up or down as desired while keeping the final concentrations of all components the same. The concentration of the target protein can be adjusted as needed; we have had success with concentrations ranging from 10 µM to 100 µM.
2 Incubate the reaction mixture at 25°C for 2–16 hr.
  The reaction time is specific to each protein. While proteins with an accessible TeCH tag may be fully labeled within 2 hr, more challenging target proteins may require 16 hr.
3 Analyze the progress of the reaction by LC‐MS.
  The progress of the reaction can be analyzed using intact‐protein LC‐MS on an Agilent 6230 TOF MS system. Given the low molecular weight of nucleophiles such as Mesna that are typically used, other assays may be challenging, as the mass shift is generally too small to detect by gel‐shift assays.
4 *Optional*: Remove excess ATP, Mesna, and TCEP by desalting on a 0.5‐mL Zeba Spin Desalting Column, 7K MWCO.
  Other methods of desalting, such as dialysis or several rounds of concentration and dilution, may also be used. Optionally, other purification steps, such as removal of His‐tagged MccB using Ni‐NTA resin, may be performed depending on the requirements of downstream applications.
5 *Optional*: Add a second thiol nucleophile (such as 4‐mercaptophenylacetic acid, MPAA) to the solution obtained after step 4 to a final concentration of 5 mM to allow *trans*‐thioesterification to occur.
  This step may be used to synthesize a C‐terminal thioester using a thiol that cannot directly capture the MccB‐generated O‐AMPylated intermediate. If step 4 is omitted, a higher concentration of the second thiol (20 mM) should be used to effect trans‐thioesterification.
6 *Optional*: Repeat step 4 to remove excess reagents and exchange the protein C‐terminal thioester into a suitable buffer for downstream applications.

## Alternate Protocol 1 MccB‐CATALYZED EXPRESSED PROTEIN LIGATION

**Figure 3:**
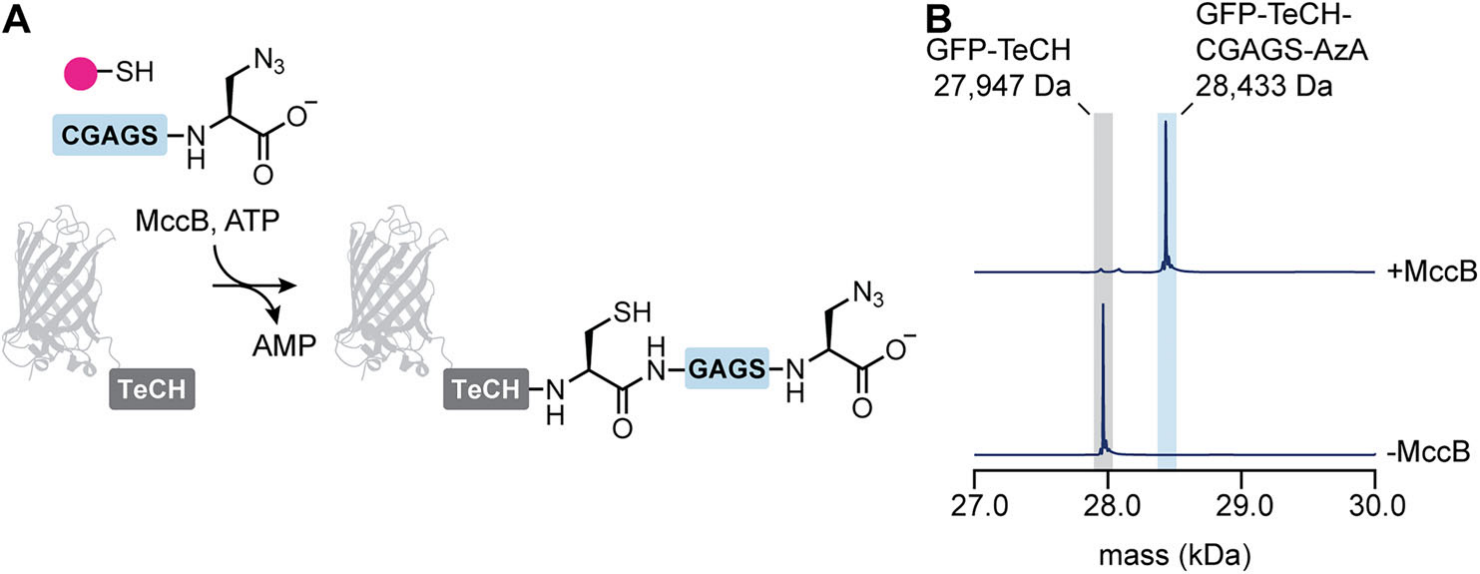
Example data for MccB‐catalyzed expressed protein ligation. (A) Reaction scheme for MccB‐catalyzed expressed protein ligation to the peptide CGAGS‐azidoA (AzA). (B) TeCH‐tagged GFP (bottom) is converted to GFP‐TeCH‐CGAGS‐AzA (top) as shown by intact protein high‐resolution LC‐MS.

The protein thioester formed between a TeCH‐tagged target protein and a small‐molecule thiol in the presence of MccB can undergo native chemical ligation when the reaction is performed in the presence of a peptide with Cys at the N‐terminus (Dawson et al., 1994; Frazier et al., 2025; Muir et al., 1998). Following protein thioester formation, *trans*‐thioesterification can occur between the C‐terminal thioester and the side‐chain thiol of the peptide's N‐terminal Cys residue. Irreversible *S*‐to‐*N* acyl shift then leads to the formation of a peptide bond. This protocol describes the procedure for carrying out one‐pot MccB‐enabled expressed protein ligation to form a peptide bond between the target protein and a synthetic peptide, without isolating the C‐terminal protein thioester. Near‐complete conversion of the target protein to the ligated product is typically achieved with this protocol (**Figure** **3**).

### Additional Materials

Peptide bearing an N‐terminal cysteine (synthesized in‐house or obtained from a commercial peptide synthesis vendor)

1 Prepare the reagents needed for step 1 in Basic Protocol 1.
2 Prepare a 50 mM stock solution of the N‐terminal Cys peptide in an appropriate solvent.
  The choice of solvent depends on the solubility of the specific N‐terminal Cys peptide to be used. Aqueous buffers are preferred, but some peptide sequences are only soluble in organic solvents such as DMSO or DMF. For these peptides, it is critical to use a concentrated stock solution (50–100 mM) to ensure a final peptide concentration of 5 mM while keeping the final DMSO/DMF concentration <10% (v/v). Higher concentrations of DMSO/DMF are incompatible with MccB activity. Alternate Protocol 2 provides a method for stepwise C‐terminal thioesterification of the target protein, followed by expressed protein ligation, which may be suitable when a higher concentration of organic solvent is required.
3 Prepare the reaction mixture according to the table below, initially omitting MccB.
  **Table:** 

**Component**	**Stock concentration**	**Final concentration**	**Vol. for a 100 µL reaction**
Reaction buffer	10×	1×	10 µL
Mesna	500 mM	5 mM	1 µL
TCEP (optional)	1 M	5 mM	0.5 µL
N‐terminal Cys peptide	50 mM	5 mM	10 µL
Target protein	100 µM	50 µM	50 µL
ATP	100 mM	5 mM	5 µL
MccB	100 µM	5 µM	5 µL
Milli‐Q water	–	–	18.5 µL
  TCEP helps to maintain Mesna in its reduced form but is optional. Mix each component thoroughly by pipetting up and down after each step. While 100 µL is a typical reaction volume, the reaction can be scaled up or down as desired while keeping the final concentrations of all components the same. The concentration of the target protein can be adjusted as needed; we have had success with concentrations ranging from 10 µM to 100 µM. If an MccB homolog other than E. coli MccB is used, the Mesna concentration should be increased to 15–25 mM.
4 Spot 0.5–1 µL of the reaction mixture on a pH indicator strip to confirm that the reaction pH is around 8.0.
  This step is critical if synthetic peptides are made or purified as trifluoroacetic acid (TFA) salts. Peptide stock solutions can sometimes be acidic, which can affect the pH of the reaction mixture. The reaction mixture must be around 8.0 for efficient MccB activity and trans‐thioesterification.
5 Initiate the reaction by adding 100 µM MccB to a final concentration of 5 µM as described in the table in step 3.
6 Incubate the reaction mixture at 25°C for 4–16 hr.
  The reaction time is specific to each protein. While proteins with an accessible TeCH tag may be fully labeled within 4 hr, more challenging target proteins may require 16 hr. The non‐enzymatic trans‐thioesterification step is generally slower than the formation of the C‐terminal thioester.
7 Analyze the progress of the reaction by LC‐MS or another assay method.
  The progress of the reaction can be analyzed using intact‐protein LC‐MS on an Agilent 6230 TOF MS system. Depending on the expected mass shift introduced by peptide ligation, other methods, such as gel‐shift assays, may be suitable for monitoring reaction progress.
8 *Optional*: Remove excess reagents and exchange the ligated protein into a suitable buffer for downstream applications by desalting on a 0.5‐mL Zeba Spin Desalting Column, 7K MWCO.
  Other methods of desalting, such as dialysis or several rounds of concentration and dilution, may also be used. The effectiveness of the chosen strategy for removing excess N‐terminal Cys peptide will depend on its ability to separate a protein of the target protein's molecular weight from a peptide of the N‐terminal Cys peptide's molecular weight. Optionally, other purification steps, such as removal of His‐tagged MccB using Ni‐NTA resin, may be performed depending on the requirements of downstream applications.

## Alternate Protocol 2 STEPWISE MccB‐CATALYZED PROTEIN C‐TERMINAL THIOESTERIFICATION FOLLOWED BY EXPRESSED PROTEIN LIGATION

In some cases, it may be desirable to perform MccB‐enabled expressed protein ligation as a stepwise process in which the target C‐terminal protein thioester is isolated and then subjected to non‐enzymatic ligation to a peptide bearing an N‐terminal Cys residue. Examples in which this approach might be useful include cases where it is desirable to make several different conjugates to the same target protein in which the sequence of the peptide to be ligated is varied, or when low solubility of the N‐terminal Cys peptide necessitates use of a high concentration of organic solvent that is not compatible with MccB activity. This protocol describes the steps for stepwise protein C‐terminal thioesterification followed by native chemical ligation. Application of this protocol typically results in near‐quantitative conversion of the target protein to the desired protein–peptide conjugate.

### Additional Materials

Peptide bearing an N‐terminal cysteine (synthesized in‐house or obtained from a commercial peptide synthesis vendor)

1 Perform steps 1–3 and optionally step 4 of Basic Protocol 1.
2 Prepare a reaction mixture containing 25–100 µM thioesterified target protein and 5 mM N‐terminal Cys peptide in a buffer at ≥ pH 8.0.
  Maintaining the pH of the reaction mixture at or above 8.0 is critical to ensure a sufficient thiolate concentration for the trans‐thioesterification reaction to proceed.
3 Incubate the reaction mixture at 25°C for 4–16 hr.
  The reaction time is specific to each protein. While proteins with an accessible TeCH tag may be fully labeled within 4 h, more challenging target proteins may require 16 hr.
4 Analyze the progress of the reaction by LC‐MS or another assay method.
  The progress of the reaction can be analyzed using intact‐protein LC‐MS on an Agilent 6230 TOF MS system. Depending on the expected mass shift introduced by peptide ligation, other methods, such as gel‐shift assays, may be suitable for monitoring reaction progress.
5 *Optional*: Remove excess reagents and exchange the ligated protein into a suitable buffer for downstream applications by desalting on a 0.5‐mL Zeba Spin Desalting Column, 7K MWCO.
  Other methods of desalting, such as dialysis or several rounds of concentration and dilution, may also be used. The effectiveness of the chosen strategy for removing excess N‐terminal Cys peptide will depend on its ability to separate a protein of the target protein's molecular weight from a peptide of the N‐terminal Cys peptide's molecular weight. Optionally, other purification steps, such as removal of His‐tagged MccB using Ni‐NTA resin, may be performed depending on the requirements of downstream applications.

## Support Protocol 1 EXPRESSION AND PURIFICATION OF MccB

**Figure 4:**
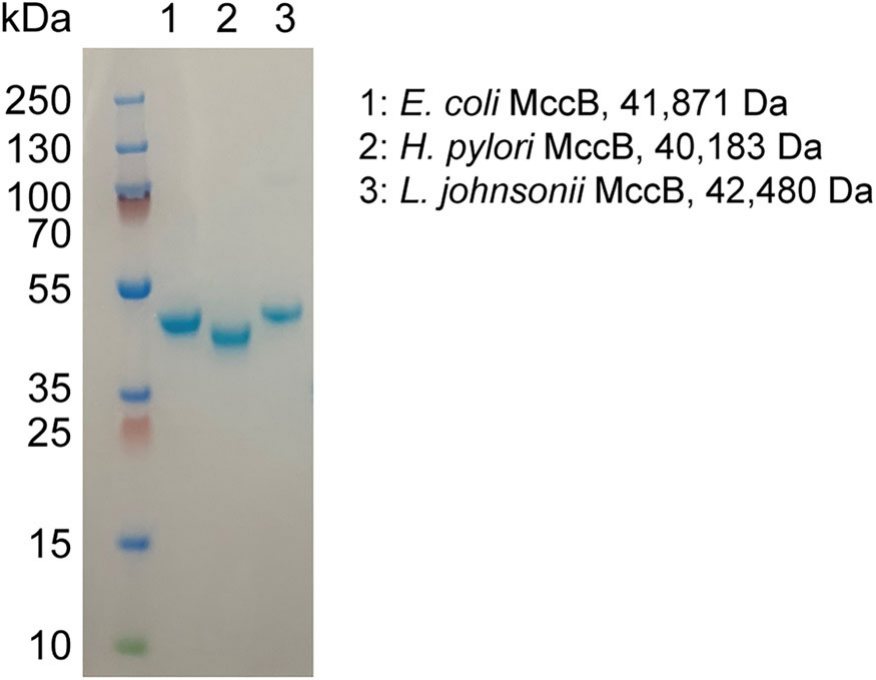
SDS‐PAGE analysis of purified MccBs from *E. coli*, *H. pylori*, and *L. johnsonii*. Left lane, PageRuler Plus Prestained protein ladder; Lane 1, *E. coli* MccB; Lane 2, *H. pylori* MccB; Lane 3, *L. johnsonii* MccB.

This Support Protocol describes the steps for expression and purification of His‐tagged *E. coli* MccB (Frazier et al., 2025). The MccB gene is modified with an N‐terminal His_6_ tag followed by a TEV protease cleavage site and is under the control of a T7 promoter. The protein is expressed in BL21 (DE3) cells by induction with isopropyl β‐D‐1‐thiogalactopyranoside (IPTG) and is purified using Ni‐NTA affinity chromatography. The same protocol, with modifications where noted, can be applied to purify MccB homologs from species other than *E. coli*. If applied properly, this protocol is expected to yield >10 mg *E. coli* MccB at >95% purity from 1 L of *E. coli* culture, with somewhat lower yields expected from other MccB homologs (**Figure** **4**).

### Materials

Luria‐Bertani (LB) broth (see recipe)
LB‐Agar plates (see recipe)
*E. coli* BL21 (DE3) chemically competent cells (Sigma‐Aldrich, cat. no. 69450‐3)
pBH4‐MccB (MccB expression plasmid; available from Addgene or upon request from A.M. Weeks)
pGro7 chaperone plasmid (Takara Bio, cat. no. 3340)
50 mg/mL Carbenicillin (see recipe)
25 mg/mL Chloramphenicol (see recipe)
1 M Isopropyl β‐D‐1‐thiogalactopyranoside (IPTG) (see recipe)
L‐(+)‐Arabinose (Sigma‐Aldrich, cat. no. A91906‐100G‐A)
Roche cOmplete, Mini, EDTA‐free Protease Inhibitor Cocktail (Sigma‐Aldrich, cat. no. 11836170001)
MccB lysis buffer (see recipe)
MccB wash buffer (see recipe)
MccB elution buffer (see recipe)
MccB storage buffer (see recipe)
PageRuler Plus Prestained Protein Ladder, 10–250 kDa (ThermoFisher Scientific, cat. no. 26619)

Glass culture tubes to accommodate 10 mL *E. coli* culture
Baffled culture flask (e.g., 2.8 L Fernbach flask, Sigma‐Aldrich, cat. no. CLS44242XL)
Refrigerated shaker incubator equipped with clamps to accommodate culture flasks (e.g., New Brunswick Innova 44R)
Refrigerated centrifuge (maximum speed >10,000 × *g*)
50‐mL conical tubes (e.g., Corning Falcon centrifuge tubes, Sigma‐Aldrich, cat. no. CLS352070)
Spectrophotometer suitable for measuring culture optical density at 600 nm (OD_600_)
Disposable plastic cuvettes for spectrophotometer
Benchtop homogenizer (e.g., Avestin Emulsiflex C3)
Ni‐NTA resin (e.g., HisPur Ni‐NTA resin, ThermoFisher Scientific, cat. no. 88221)
Disposable chromatography column (e.g., Bio‐Spin Chromatography Columns, Bio‐Rad, cat. no. 7326008)
Laboratory rocker
Microvolume spectrophotometer (such as a Nanodrop microvolume spectrophotometer)
Additional reagents and equipment for SDS‐PAGE (Gallagher, 2012)
10,000 MWCO dialysis tubing (e.g., SnakeSkin dialysis tubing, ThermoFisher Scientific, cat. no. 68100)

1 Transform *E. coli* BL21(DE3) with pBH4‐MccB (and pGro7, if required; see Support Protocol 1 Introduction) using standard heat shock or electroporation protocols appropriate for the competent cells used.
  For MccB from organisms other than E.coli, E.coli BL21(DE3) should be co‐transformed with pGro7 to improve yield and solubility. Co‐transformation with pGro7 has little impact on the yield of E. coli MccB.
2 *Optional*: Add 1 mL LB broth and allow the cells to recover with shaking for 1 hr at 37°C.
  This step is necessary only if pGro7 was simultaneously transformed with pBH4‐MccB.
3 After transformation, plate the cells on an LB agar plate supplemented with the appropriate antibiotics.
  If only pBH4‐MccB was used, the plate should be supplemented with 50 µg/mL carbenicillin. If both pBH4‐MccB and pGro7 were transformed, the plate should be supplemented with 50 µg/mL carbenicillin and 25 µg/mL chloramphenicol.
4 Incubate the LB agar plate at 37°C overnight (∼16 hr) until single colonies grow.
5 Place 10 mL LB supplemented with the appropriate antibiotics in a glass culture tube and inoculate with a single colony from step 4.
6 Incubate the 10‐mL culture at 37°C overnight (∼16 hr) with shaking at 200 rpm.
7 The next day, place 1 L LB broth in a 2.8‐L Fernbach flask and inoculate with the 10 mL starter culture from step 6.
8 Grow the expression culture at 37°C with shaking at 200 rpm. Measure the OD_600_ every 30 min until it reaches 0.6–0.8.
9 When the culture reaches OD_600_ 0.6–0.8, chill it on ice for 20 min.
10 While the culture is chilling, cool a refrigerated shaker incubator to 18°C for protein expression.
11 Induce protein expression by adding IPTG (1 M) to a final concentration of 400 µM. For example, for a 1‐L culture, 400 µL of 1 M IPTG should be added.
  If pGro7 has also been transformed, induce expression by adding IPTG to a final concentration of 0.25 mM and also add solid arabinose to a final concentration of 2 g/L.
12 Incubate the expression culture at 18°C with shaking at 200 rpm for 16–20 hr to allow protein expression to proceed.
13 Harvest the cells by centrifugation at 4000 × *g* for 20 min at 4°C. Discard the supernatant and collect the pellet.
  The pellet can be saved at −80°C until ready for purification.
14 Resuspend the cell pellet in 35 mL MccB lysis buffer supplemented with one Complete Mini EDTA‐free protease inhibitor cocktail tablet. Mix the cells until homogeneous and free of clumps before proceeding to the next step.
15 Lyse the cells by passing the cell suspension through a benchtop homogenizer at 15,000 psi.
  Other lysis methods, such as sonication, may be used if a benchtop homogenizer is not available.
16 Repeat step 15 two additional times.
17 Clear the supernatant by centrifugation for 30 min at 10,000 × *g* and 4°C. Transfer the MccB‐containing supernatant into a new 50‐mL conical tube and keep on ice.
18 Place 2 mL Ni‐NTA resin slurry in a disposable chromatography column. Wash the resin with 10 mL water.
19 Wash the resin with 10 mL MccB lysis buffer.
20 Resuspend the Ni‐NTA resin with 1 mL MccB lysis buffer and transfer to a 50‐mL conical tube.
21 Add the supernatant from step 17 to the resin and incubate for 30 min to 1 hr at 4°C with gentle rocking on a laboratory rocker.
  Other gentle mixing devices, such as an end‐over‐end mixer, are also suitable.
22 Pellet the resin by centrifugation for 3 min at 500 × *g* and 4°C. Pour off the supernatant without disturbing the resin.
23 Resuspend the resin in MccB wash buffer and transfer back to the same chromatography column used in step 18.
24 Wash the resin with 10–20 column volumes (10–20 mL) of MccB wash buffer by gravity flow.
25 Elute the protein with 3 column volumes (3 mL) of MccB elution buffer. Monitor the absorbance of the eluent at 280 nm using a microvolume spectrophotometer to ensure that protein elution is complete.
26 Dialyze eluted protein into MccB storage buffer.
27 After dialysis, determine the concentration of MccB by measuring the absorbance at 280 nm (ϵ = 52,300 M^−1^ cm^−1^ based on ProtParam analysis).
28 Analyze the protein by SDS‐PAGE to assess its purity. Load 5 µg purified protein into one well of a 10‐well 4% to 20% mini‐gel. In an adjacent well, load 5 µL of PageRuler Plus Prestained 10–250 kDa protein ladder or a similar protein ladder.
  Typical purity is >95%.
29 Adjust the final concentration to 100 µM by diluting in MccB storage buffer or concentrating. Prepare single‐use aliquots, flash freeze in liquid nitrogen, and store at −80°C.

## Basic Protocol 2 MccB‐ AND SUBTILIGASE‐CATALYZED EXPRESSED PROTEIN LIGATION

**Figure 5:**
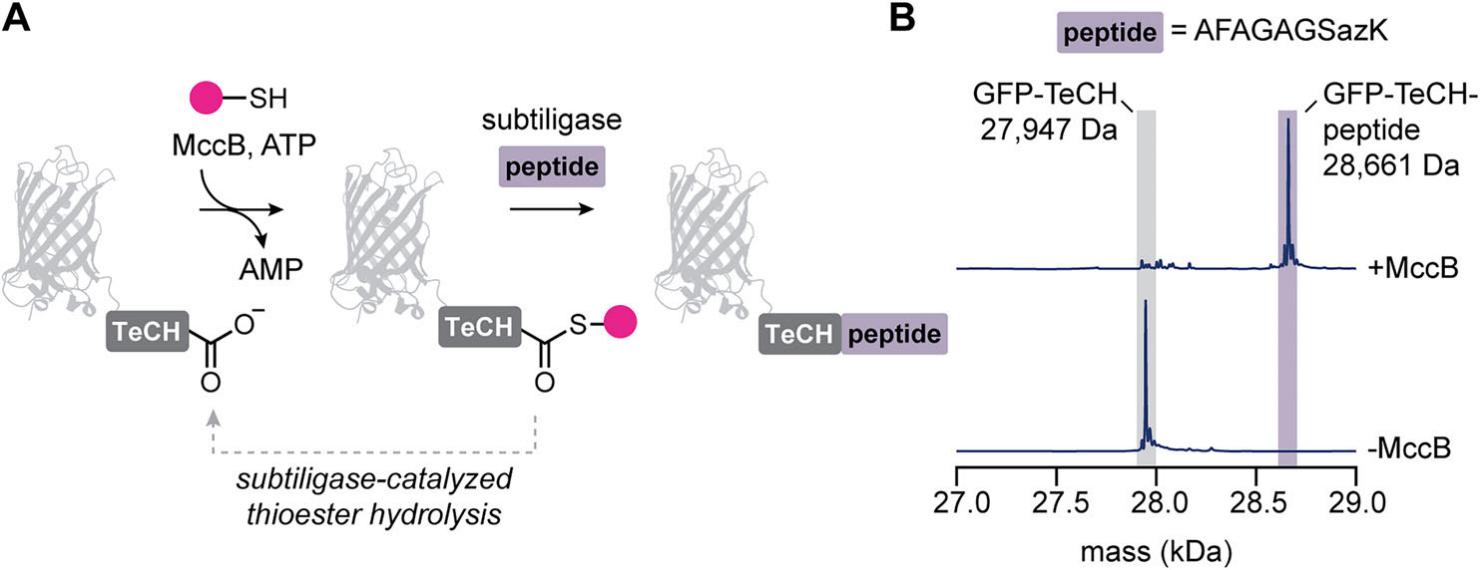
Example data for MccB‐ and subtiligase‐catalyzed expressed protein ligation. (A) Reaction scheme for MccB‐ and subtiligase‐catalyzed expressed protein ligation to the peptide AFAGAGS‐azidoK. (B) TeCH‐tagged GFP (bottom) is converted to GFP‐TeCH‐AFAGAGS‐azidoK (top) as shown by intact protein high‐resolution LC‐MS.

Enzyme‐catalyzed expressed protein ligation is a variation of EPL that eliminates the need for Cys at the ligation junction by using subtiligase to catalyze ligation of alternative peptide sequences to a protein C‐terminal thioester (Henager et al., 2016). Subtiligase catalyzes the formation of a peptide bond between a C‐terminal ester/thioester donor and the N‐terminal α‐amine of an acceptor peptide (Weeks & Wells, 2020b). Subtiligase has broad N‐terminal specificity, and different variants have been engineered to expand the scope of sequences that are subtiligase substrates (Weeks & Wells, 2018, 2020a). However, subtiligase‐catalyzed hydrolysis (rather than ligation) of the C‐terminal thioester can limit ligation yield, especially when the C‐terminal thioester has been generated by methods that do not allow for thioester regeneration. Synthesis of the protein C‐terminal thioester *in situ* using MccB‐catalyzed protein thioesterification can improve reaction yields by enabling ATP‐driven thioester regeneration. This method is suitable for use when it is acceptable to have the TeCH tag sequence at the ligation junction. Basic Protocol 2 describes the application of MccB and subtiligase for enzyme‐catalyzed EPL. Execution of this protocol is expected to yield near‐complete conversion of a C‐terminally TeCH‐tagged protein and synthetic subtiligase substrate peptide to the ligation product (**Figure** **5**).

### Materials

10× Reaction buffer (see recipe)
100 mM Adenosine 5′‐triphosphate (ATP), pH 7.0 (see recipe)
1 M Tris(2‐carboxyethyl)phosphine (TCEP), pH 8.0 (see recipe)
Ultrapure Milli‐Q water
500 mM Sodium 2‐mercaptoethanesulfonate (Mesna), pH 7.0 (prepare fresh; see recipe)
100 µM MccB (see Support Protocol 1)
100 µM Subtiligase (Weeks & Wells, 2020a)
C‐terminally TeCH‐tagged target protein (see Strategic Planning)
Synthetic peptide to be ligated to TeCH‐tagged protein C‐terminus (see Strategic Planning)
pH indicator strips (pH 0–14, Sigma‐Aldrich, cat. no. 1095350001)
LC‐MS system suitable for intact protein analysis (e.g., Agilent 1290 LC system coupled to Agilent 6230 time‐of‐flight (TOF) mass spectrometer)
Analytical HPLC column suitable for intact protein analysis on the LC‐MS system (e.g., Agilent PLRP‐S 1000Å, 2.1 × 50 mm, 5 µm, cat. no. PL1912‐1502)
Software capable of deconvolution of multiply charged intact protein mass spectra (e.g., Agilent BioConfirm, Maximum Entropy algorithm)

1 Prepare a 50 mM stock solution of the peptide to be ligated to the TeCH‐tagged protein C‐terminus in an appropriate solvent.
  The choice of solvent depends on the solubility of the specific synthetic peptide to be used. Aqueous buffers are preferred, but some peptide sequences are only soluble in organic solvents such as DMSO or DMF. For these peptides, it is critical to use a concentrated stock solution (50–100 mM) to ensure a final peptide concentration of 5 mM while keeping the final DMSO/DMF concentration <10% (v/v). Higher concentrations of DMSO/DMF are incompatible with MccB activity and may also affect subtiligase activity.
2 Prepare the reaction mixture according to the table below, initially omitting MccB and subtiligase.
  **Table:** 

**Component**	**Stock concentration**	**Final concentration**	**Vol. for a 100 µL reaction**
Reaction buffer	10×	1×	10 µL
Mesna	500 mM	5 mM	1 µL
TCEP (optional)	1 M	5 mM	0.5 µL
Synthetic peptide	50 mM	5 mM	10 µL
Target protein	100 µM	50 µM	50 µL
ATP	100 mM	5 mM	5 µL
MccB	100 µM	5 µM	5 µL
Subtiligase	100 µM	5 µM	5 µL
Milli‐Q water	–	–	13.5 µL
  For enzyme‐catalyzed expressed protein ligation, it is critical to avoid buffers with primary amines, such as Tris and glycerol. When these molecules are present, they can be ligated to the C‐terminus of the TeCH‐tagged protein in a subtiligase‐dependent manner. TCEP helps to maintain Mesna in its reduced form but is optional. Mix each component thoroughly by pipetting up and down after each step. While 100 µL is a typical reaction volume, the reaction can be scaled up or down as desired, keeping the final concentrations of all components unchanged. The concentration of the target protein can be adjusted as needed; we have had success with concentrations ranging from 10 µM to 100 µM.
3 Spot 0.5–1 µL of the reaction mixture on a pH indicator strip to confirm that the reaction pH is around 8.0.
  This step is critical if synthetic peptides are made or purified as trifluoroacetic acid (TFA) salts. Peptide stock solutions can sometimes be acidic, which can affect the pH of the reaction mixture. The reaction mixture must be around 8.0 for efficient MccB and subtiligase activity.
4 Add 100 µM subtiligase to a final concentration of 5 µM as indicated in the table in step 2.
5 Add 100 µM MccB to a final concentration of 5 µM to initiate the reaction as indicated in the table in step 2.
6 Incubate the reaction mixture at 25°C for 2–16 hr.
  The reaction time is specific to each TeCH‐tagged protein and synthetic peptide. While proteins with an accessible TeCH tag may be fully labeled within 4 hr, more challenging target proteins may require 16 hr.
7 Analyze the progress of the reaction by LC‐MS or another assay method.
  The progress of the reaction can be analyzed using intact‐protein LC‐MS on an Agilent 6230 TOF MS system. Depending on the expected mass shift introduced by peptide ligation, other methods, such as gel‐shift assays, may be suitable for monitoring reaction progress.
8 *Optional*: Remove excess reagents and exchange the ligated protein into a suitable buffer for downstream applications by desalting on a 0.5‐ml Zeba Spin Desalting Column, 7K MWCO.
  Other methods of desalting, such as dialysis or several rounds of concentration and dilution, may also be used. The effectiveness of the chosen strategy for removing excess synthetic peptide will depend on its ability to separate a protein of the target protein's molecular weight from a peptide of the synthetic peptide's molecular weight. Optionally, additional purification steps, such as removing His‐tagged MccB and His‐tagged subtiligase using Ni‐NTA resin, may be performed depending on the requirements of downstream applications.

## REAGENTS AND SOLUTIONS

Use Milli‐Q water unless otherwise specified.

### Adenosine 5′‐triphosphate (ATP), pH 7.0, 100 mM

100 mM adenosine 5′‐triphosphate (Sigma‐Aldrich, cat. no. A2383‐1G)
Carefully adjust the pH of the solution to 7.0 using 1 M NaOH; use pH strips to test
Prepare 50 µL aliquots and store at −20°C for up to a year
Avoid repeated freeze‐thaw cycles

### Carbenicillin, 50 mg/mL

500 mg carbenicillin (Gold Biotechnology, cat. no. C‐103)
Ultrapure water to 10 mL
Sterilize by passing through a 0.2‐µm filter (e.g., Sigma‐Aldrich, cat. no. CLS431222)
Aliquot and store at −20°C for up to a year

### Chloramphenicol, 25 mg/mL

250 mg chloramphenicol (Gold Biotechnology, cat. no. C‐105)
Ethanol to 10 mL
Aliquot and store at ‐20 °C for up to a year

### Isopropyl β‐D‐1‐thiogalactopyranoside (IPTG), 1 M

1 M IPTG (Gold Biotechnology, cat. no. I2481C)
Sterilize by passing through a 0.2‐µm filter (e.g., Sigma‐Aldrich, cat. no. CLS431222)
Aliquot and store at −20°C for up to a year

### LB‐agar plates

40 g LB broth with agar (Miller) (Sigma‐Aldrich, cat. no. L3147)
Ultrapure water to 1 L
Autoclave for 30 minutes at 121°C to sterilize
Cool to 55°C prior to adding appropriate antibiotics
Pour 35 mL into disposable 100‐mm Petri dishes
Store at 4°C for up to 1 month

### Luria‐Bertani (LB) broth

25 g LB broth (Miller) (Sigma‐Aldrich, cat. no. L3522)
Ultrapure water to 1 L
Autoclave for 30 minutes at 121°C to sterilize

### MccB elution buffer

25 mM Tris·HCl, pH 8.0
500 mM NaCl
10 mM MgCl_2_
200 mM imidazole
Store at room temperature for up to a year.

### MccB lysis buffer

25 mM Tris·HCl, pH 8.0
500 mM NaCl
10 mM MgCl_2_
Store at room temperature for up to a year

### MccB storage buffer

25 mM Tris·HCl, pH 8.0
500 mM NaCl
1 mM DTT
10% (v/v) glycerol
Store at 4 °C for up to a week

The glycerol should be omitted if MccB will be used for enzyme‐catalyzed expressed protein ligation, as subtiligase will catalyze C‐terminal esterification with glycerol

### MccB wash Buffer

25 mM Tris·HCl, pH 8.0
500 mM NaCl
10 mM MgCl_2_
25 mM imidazole
Store at room temperature for up to a year

### Reaction buffer, 10×

750 mM Tris·HCl, pH 8.0 or 750 mM HEPES, pH 8.0
50 mM MgCl_2_
For a 10 mL stock, add 0.909 g Tris base or 1.79 g HEPES and 1.017 g MgCl_2_•6H_2_O to 8 mL nuclease‐free water. Adjust pH to 8.0 with HCl (for Tris buffer) or NaOH (for HEPES buffer), then bring to a final volume of 10 mL.
Filter‐sterilize and store at 4°C for up to two weeks

### Sodium 2‐mercaptoethanesulfonate (Mesna), pH 7.0, 500 mM

500 mM sodium 2‐mercaptoethanesulfonate (Sigma‐Aldrich, cat. no. M1511‐5G)
Carefully adjust the pH to 7.0 using 1 M NaOH; use pH strips to monitor the pH
Prepare fresh or prepare single‐use aliquots and store at −80°C for up to 1 month

### Tris(2‐carboxyethyl)phosphine (TCEP), pH 8.0, 1 M

1 M TCEP (Gold Biotechnology, cat. no. TCEP1)
Carefully adjust the pH to 8.0 using 5 M NaOH; pH adjustment will aid in the dissolution of TCEP
Prepare 100 µL aliquots and store at −20°C for up to a year

## COMMENTARY

### Background Information

Originally discovered in the biosynthetic gene cluster for the RiPP natural product microcin C7, MccB specifically recognizes its heptapeptide substrate MccA and converts the C‐terminal Asn to iso‐Asn‐AMP phosphoramidate using ATP as a co‐substrate (Guijarro et al., 1995; Roush et al., 2008). The reaction proceeds through a C‐terminally *O*‐AMPylated intermediate, which is intramolecularly captured by the C‐terminal Asn side chain in the native reaction. We recently found that if the C‐terminal Asn residue is substituted with Gly, this non‐native substrate can still be activated by *O*‐AMPylation (Frazier et al., 2025). The resultant *O*‐AMPylated peptide mixed anhydride is a reactive electrophile that can be efficiently captured by thiol nucleophiles to form a C‐terminal peptide or protein thioester. These peptide/protein C‐terminal thioesters are attractive bioconjugation intermediates as they are kinetically stable but thermodynamically activated electrophiles that can be used in downstream reactions.

The small size of the MccA substrate peptide and the high specificity of MccB for its cognate substrate enable the development of the MccB/TeCH tag system for C‐terminal protein thioesterification. Key to this system is the fusion of an engineered MccA sequence (MccA‐N7G, the TeCH tag) to the C‐terminus of the target protein, enabling ATP‐driven, MccB‐catalyzed C‐terminal thioesterification. These MccB‐generated thioesters can be used in a variety of bioconjugation processes, including NCL, EPL, and enzyme‐catalyzed EPL. Importantly, in the case of MccB/subtiligase‐catalyzed EPL, MccB‐catalyzed regeneration of thioesters from hydrolyzed side products results in improved yield compared to other thioester generation methods that are commonly used for EPL.

Profiling the natural diversity of the MccA/MccB system has expanded the available toolbox of MccB/TeCH tag pairs (Frazier et al., 2025). Highly specific MccBs from *E. coli*, *L. johnsonii*, and *H. pylori* have been used for epitope‐specific labeling of target proteins with the corresponding TeCH tags, highlighting the orthogonality of these tools. On the other hand, more sequence‐tolerant MccBs, such as *H. somni* MccB, have been used for the synthesis of ubiquitin‐derived peptide thioesters that are substrates for lysine acylation using the conjugating enzymes (LACE) system for lysine modification (Frazier et al., 2025). Together, MccB/TeCH tag methods are broadly useful for driving bioconjugation reactions to high yield by coupling peptide bond formation to ATP cleavage and can be integrated with existing chemical biology tools to enable innovation in protein modification for probing and manipulating biology.

### Critical Parameters

#### Reaction pH

Reaction pH is important for maintaining optimal enzyme activity and ensuring a sufficient concentration of deprotonated thiol for non‐enzymatic reactions. For Basic Protocols 1 and 2, adjust the pH of any acidic stock solutions to 7 to 8 before adding them to the reaction mixture, and set the final reaction pH to 8.0, with an acceptable range of 8.0 to 8.5. Best practice is to check the pH of the reaction mixture with a pH indicator strip before adding the enzymes.

#### Nucleophile size and structure

The ability of the thiol nucleophile to access the enzyme active site to capture the C‐terminal *O*‐AMP intermediate is critical. We have found that Mesna functions optimally for this purpose. For larger nucleophiles, *trans*‐thioesterification of the protein‐Mes thioester can be performed after the enzymatic reaction. To ensure that the nucleophile is present in its reduced form, it is best to prepare the nucleophile stock solution fresh.

#### Target protein/peptide sequence and structure

To enable efficient MccB‐catalyzed thioesterification, the target protein must be modified with a structurally accessible C‐terminal TeCH tag (see Strategic Planning). It is advisable to analyze the TeCH‐tagged target protein using high‐resolution intact‐protein LC‐MS to ensure that unexpected cleavage of the tag has not occurred during expression or purification. For MccB/subtiligase‐catalyzed EPL, the N‐terminal sequence of the acceptor peptide to be ligated must be matched with a subtiligase variant that modifies that sequence efficiently.

### Troubleshooting Table

See Table 1.

**Table 1:** Troubleshooting Guide for MccB‐catalyzed C‐terminal Protein Modification

**Problem**	**Possible Cause**	**Solution**
Low conversion to thioester	Thiol nucleophile cannot access is the active site of MccB	Form Mes thioester and then use *trans*‐thioesterification strategy to introduce desired thiol
	Reaction pH is too low	Check the pH and adjust to 8.0 prior to addition of MccB
	Thiol nucleophile has oxidized	Prepare a fresh stock of thiol nucleophile or add an equal concentration of TCEP to the reaction
	TeCH tag on target protein is inaccessible to MccB	Add a short, flexible linker between target protein and TeCH tag (see Strategic Planning)
	TeCH tag on target protein has been proteolyzed	Analyze the exact mass of purified target protein prior to beginning to ensure TeCH tag is intact
		Use protease inhibitors during target protein purification and take care to keep sample at 4 °C during purification to minimize protease activity
		Use alternative TeCH tag sequence from a different bacterial species (see Strategic Planning
Low yield from enzyme‐catalyzed expressed protein ligation	Low thioester formation due to inefficiency of MccB. Diagnose by performing test reactions in the presence of MccB and the absence of subtiligase and analyzing target protein conjugates by LC‐MS. If little thioester is formed in the absence of subtiligase, low MccB efficiency is the likely cause	See ‘low conversion to thioester’ above
	Low thioester formation due to subtiligase‐catalyzed thioester hydrolysis. Diagnose by comparing thioester formation in the presence and absence of subtiligase. If thioester is formed efficiently in the presence of MccB only but neither ligated product nor thioester are observed in the presence of subtiligase, subtiligase‐catalyzed hydrolysis is the likely cause	Change the sequence of the C‐terminal side of the ligation junction to better match subtiligase specificity (Weeks & Wells, 2020a)
		Use a subtiligase variant that more efficiently accepts the desired acceptor peptide sequence (Weeks & Wells, 2020a)
	Subtiligase‐catalyzed formation of unexpected C‐terminal target protein conjugates	Avoid nucleophiles other than Mesna in reaction buffer, especially Tris and glycerol
	Sequence of subtiligase acceptor peptide is not ideal for the subtiligase variant being used	Change the sequence of the C‐terminal side of the ligation junction to match subtiligase specificity (Weeks & Wells, 2020a)
		Use a subtiligase variant that more efficiently accepts the desired acceptor peptide sequence (Weeks & Wells, 2020a)
Unexpected protein masses observed by LC‐MS	Truncation/proteolysis of target protein during expression and purification	Use protease inhibitors during purification
	Nucleophiles other than Mesna/desired thiol present in reaction mixture	Avoid nucleophiles in reaction buffer and enzyme/target protein storage buffers (e.g., Tris, glycerol)

### Understanding Results

Example results for Basic Protocol 1 are shown in **Figure** **2**. For a target protein modified with an accessible C‐terminal TeCH tag, near‐quantitative conversion to the Mes thioester is expected within 16 hr when Mesna is provided as the thiol nucleophile. For target proteins with a less accessible TeCH tag, lower yields are expected. Conversion of the TeCH‐tagged protein to the thioester is assessed by intact‐protein high‐resolution LC‐MS. A mass shift corresponding to the formation of the thioester is observed when the reaction is successful. When the optional *trans*‐thioesterification step in Basic Protocol 1 is performed, conversion to both the Mes and MPAA thioesters can be assessed by high‐resolution LC‐MS.

Example results for Alternative Protocols 1 and 2 are shown in **Figure** **3**. Performing the C‐terminal thioesterification reaction in the presence of an N‐terminal Cys peptide results in the formation of a C‐terminal Mes thioester, *trans*‐thioesterification with the N‐terminal Cys thiol, and an *S*‐to‐*N* acyl shift to form a peptide bond. Formation of the C‐terminal Mes thioester intermediate and the ligated product is monitored by intact‐protein high‐resolution LC‐MS.

Expected results from Support Protocol 1 are shown in **Figure** **4**. MccB purity was assessed by SDS‐PAGE and was judged to be >95%. Purification of *E. coli* MccB from 1 L of *E. coli* culture is expected to yield >10 mg of protein. Other MccB homologs produce lower yield (1–5 mg/L).

A typical result for MccB/subtiligase‐catalyzed expressed protein ligation (Basic Protocol 2) is shown in **Figure** **5**. A mass shift corresponding to the formation of an amide bond between the TeCH‐tagged protein and the acceptor peptide, as well as the mass shift corresponding to the formation of the thioester intermediate, can be observed by intact protein high‐resolution LC‐MS. Common problems encountered in Basic Protocol 2 can typically be diagnosed using HR‐LC‐MS. For the ligation reaction to occur successfully, the target protein must have an accessible C‐terminal TeCH tag and a suitable acceptor peptide N‐terminal sequence compatible with recognition by subtiligase. If problems are encountered, it is advisable to assess the ability of the TeCH‐tagged protein to be converted to the C‐terminal thioester by MccB in the absence of subtiligase to pinpoint which enzyme is not working efficiently under the reaction conditions. We have observed that subtiligase can catalyze ligation of undesired nucleophiles, such as Tris or glycerol, to the C‐terminus of TeCH‐tagged target proteins. In the event that an unexpected mass increase is observed, the composition of each component solution, including enzyme and target protein storage buffers, should be carefully evaluated for the presence of unanticipated ligation partners. We have also observed that, for certain target proteins, MccB and subtiligase can catalyze their cyclization. Typical features of such proteins include N‐ and C‐termini that are close together in the 3D structure and N‐terminal sequences that are good substrates for subtiligase. This cyclization activity can typically be suppressed by using a high concentration (5 mM) of the acceptor peptide.

### Time Considerations

Execution of Basic Protocol 1 or 2 requires approximately 2 h of hands‐on time for reagent preparation and reaction setup and about 4 to 16 hr of reaction time depending on the target protein. Purification of MccB (Support Protocol 1, required for Basic Protocols 1 and 2) requires 2 to 3 days. Purification of subtiligase (required for Basic Protocol 2; Weeks & Wells, 2020a) requires approximately 2 days. Both protein purifications are expected to yield enough material for many bioconjugation experiments. The amount of hands‐on time required for Basic Protocols 1 and 2 is significantly reduced after initial reagent preparation is completed.

### Author Contributions

**Di Yang**: Writing—original draft; conceptualization; methodology; validation; writing—review and editing. **Amy M. Weeks**: Conceptualization; writing—original draft; methodology; validation; funding acquisition; supervision.

### Conflict of Interest

The Wisconsin Alumni Research Foundation has filed a provisional patent application related to the MccB/TeCH tag system on which A.M.W. is an inventor. D.Y. declares no conflicts of interest.

## Data Availability

Data sharing is not applicable to this article as no new data were created or analyzed in this study.
